# Supplementary material for: BERT-5mC: an interpretable model for predicting 5-methylcytosine sites of DNA based on BERT
Source: PeerJ. 2023 Dec 8;11:e16600. doi: 10.7717/peerj.16600 (PMC10712318; doi:10.7717/peerj.16600)
Supplement: Supplemental Information 1 [file peerj-11-16600-s001.docx]

**Table S1.** Performance evaluation on the independent test set based on the fine-tuned models built by using different word sizes.

|  | Word size | Sen | Spe | ACC | MCC | AUROC | AUPRC |
| --- | --- | --- | --- | --- | --- | --- | --- |
| Independent test set | 1mer | 0.854 | 0.933 | 0.927 | 0.631 | 0.962 | 0.570 |
|  | 3mer | 0.872 | 0.938 | 0.933 | 0.656 | 0.966 | 0.604 |
|  | 5mer | 0.831 | 0.941 | 0.933 | 0.640 | 0.965 | 0.594 |

**Table S2**. Performance evaluation of models built by using embedding features extracted from the pre-trained Promoter-BERT model.

|  | | Classifier | Sen | Spe | ACC | MCC | AUROC | AUPRC |
| --- | --- | --- | --- | --- | --- | --- | --- | --- |
| 5-fold cross validation | bert_CLS(XGB) | 0.856 | 0.632 | 0.649 | 0.267 | 0.819 | 0.249 | |
|  | bert_CLS(RF) | 0.000 | 1.000 | 0.922 | 0.009 | 0.802 | 0.229 | |
|  | bert_CLS(FFNN) | 0.229 | 0.965 | 0.908 | 0.238 | 0.845 | 0.286 | |
|  | bert_CLS(BiLSTM) | 0.411 | 0.951 | 0.909 | 0.364 | 0.898 | 0.396 | |
|  | NPF+bert_CLS(XGB) | 0.988 | 0.824 | 0.837 | 0.511 | 0.955 | 0.548 | |
|  | NPF+bert_CLS(RF) | 0.187 | 0.987 | 0.925 | 0.292 | 0.934 | 0.464 | |
|  | NPF+bert_CLS(FFNN) | 0.692 | 0.943 | 0.924 | 0.553 | 0.953 | 0.521 | |
|  | NPF+bert_CLS(BiLSTM) | 0.849 | 0.917 | 0.912 | 0.588 | 0.957 | 0.560 | |
| Independent test | bert_CLS(XGB) | 0.863 | 0.627 | 0.646 | 0.267 | 0.820 | 0.254 | |
|  | bert_CLS(RF) | 0.000 | 1.000 | 0.922 | 0.007 | 0.803 | 0.233 | |
|  | bert_CLS(FFNN) | 0.346 | 0.945 | 0.898 | 0.292 | 0.851 | 0.303 | |
|  | bert_CLS(BiLSTM) | 0.499 | 0.939 | 0.905 | 0.402 | 0.899 | 0.526 | |
|  | NPF+bert_CLS(XGB) | 0.987 | 0.824 | 0.836 | 0.510 | 0.955 | 0.546 | |
|  | NPF+bert_CLS(RF) | 0.193 | 0.987 | 0.925 | 0.297 | 0.934 | 0.467 | |
|  | NPF+bert_CLS(FFNN) | 0.719 | 0.941 | 0.924 | 0.564 | 0.953 | 0.397 | |
|  | NPF+bert_CLS(BiLSTM) | 0.783 | 0.936 | 0.924 | 0.592 | 0.957 | 0.554 | |

**Table S3**. Performance evaluation of models built by using embedding features extracted from fine-tuned Promoter-BERT model.

|  | | Classifier | Sen | Spe | ACC | MCC | AUROC | AUPRC |
| --- | --- | --- | --- | --- | --- | --- | --- | --- |
| 5-fold cross validation | bert_CLS(XGB) | 0.967 | 0.862 | 0.870 | 0.565 | 0.963 | 0.569 | |
|  | bert_CLS(RF) | 0.955 | 0.885 | 0.891 | 0.597 | 0.962 | 0.566 | |
|  | bert_CLS(FFNN) | 0.890 | 0.929 | 0.926 | 0.645 | 0.964 | 0.569 | |
|  | bert_CLS(BiLSTM) | 0.918 | 0.920 | 0.920 | 0.639 | 0.964 | 0.574 | |
|  | NPF+bert_CLS(XGB) | 0.968 | 0.862 | 0.870 | 0.565 | 0.963 | 0.568 | |
|  | NPF+bert_CLS(RF) | 0.954 | 0.886 | 0.892 | 0.598 | 0.962 | 0.566 | |
|  | NPF+bert_CLS(FFNN) | 0.886 | 0.933 | 0.929 | 0.651 | 0.963 | 0.566 | |
|  | NPF+bert_CLS(BiLSTM) | 0.916 | 0.921 | 0.921 | 0.640 | 0.964 | 0.575 | |
| Independent test | bert_CLS(XGB) | 0.958 | 0.900 | 0.905 | 0.618 | 0.966 | 0.602 | |
|  | bert_CLS(RF) | 0.872 | 0.937 | 0.932 | 0.652 | 0.966 | 0.602 | |
|  | bert_CLS(FFNN) | 0.877 | 0.936 | 0.931 | 0.653 | 0.966 | 0.599 | |
|  | bert_CLS(BiLSTM) | 0.874 | 0.936 | 0.931 | 0.653 | 0.966 | 0.602 | |
|  | NPF+bert_CLS(XGB) | 0.958 | 0.900 | 0.905 | 0.618 | 0.966 | 0.603 | |
|  | NPF+bert_CLS(RF) | 0.872 | 0.937 | 0.932 | 0.652 | 0.966 | 0.602 | |
|  | NPF+bert_CLS(FFNN) | 0.874 | 0.936 | 0.931 | 0.652 | 0.966 | 0.601 | |
|  | NPF+bert_CLS(BiLSTM) | 0.874 | 0.936 | 0.931 | 0.652 | 0.966 | 0.602 | |

**Table S4**. Hyperparameter search range for each learning algorithm

| Learning algorithm | Hyperparameter range |
| --- | --- |
| XGBoost | Learning rate = [0.0005-0.1] |
|  | max_depth = [6,8,10] |
|  | n_estimators = [1200,1600,1800,2000] |
|  | scale_pos_weight = [5, 8] |
| Random Forest | max_depth = [6,8,10] |
|  | n_estimators = [1200,1800,2400] |
| FFNN | epoch = [16,32,64] |
|  | batch size = [16,32,64] |
|  | learning rate = [0.00001-0.001] |
| BiLSTM | epoch = [16,32,64] |
|  | batch size = [16,32,64] |
|  | learning rate = [0.00001-0.001] |

**Table S5**. Performance evaluation of models built by using average embeddings (bert_AVE) of the 39 3-mers extracted from fine-tuned Promoter-BERT model.

|  | | Classifier | Sen | Spe | ACC | MCC | AUROC | AUPRC |
| --- | --- | --- | --- | --- | --- | --- | --- | --- |
| 5-fold cross validation | bert_AVE(XGB) | 0.967 | 0.862 | 0.870 | 0.565 | 0.963 | 0.569 | |
|  | bert_AVE(RF) | 0.955 | 0.885 | 0.891 | 0.597 | 0.962 | 0.566 | |
|  | bert_AVE(FFNN) | 0.890 | 0.929 | 0.926 | 0.645 | 0.964 | 0.569 | |
|  | bert_AVE(BiLSTM) | 0.918 | 0.920 | 0.920 | 0.639 | 0.964 | 0.574 | |
|  | NPF+bert_AVE(XGB) | 0.968 | 0.862 | 0.870 | 0.565 | 0.963 | 0.568 | |
|  | NPF+bert_AVE(RF) | 0.954 | 0.886 | 0.892 | 0.598 | 0.962 | 0.566 | |
|  | NPF+bert_AVE(FFNN) | 0.886 | 0.933 | 0.929 | 0.651 | 0.963 | 0.566 | |
|  | NPF+bert_AVE(BiLSTM) | 0.916 | 0.921 | 0.921 | 0.640 | 0.964 | 0.575 | |
| Independent test | bert_AVE(XGB) | 0.958 | 0.900 | 0.905 | 0.618 | 0.966 | 0.602 | |
|  | bert_AVE(RF) | 0.872 | 0.937 | 0.932 | 0.652 | 0.966 | 0.602 | |
|  | Bert_AVE(FFNN) | 0.877 | 0.936 | 0.931 | 0.653 | 0.966 | 0.599 | |
|  | bert_AVE(BiLSTM) | 0.874 | 0.936 | 0.931 | 0.653 | 0.966 | 0.602 | |
|  | NPF+bert_AVE(XGB) | 0.958 | 0.900 | 0.905 | 0.618 | 0.966 | 0.603 | |
|  | NPF+bert_AVE(RF) | 0.872 | 0.937 | 0.932 | 0.652 | 0.966 | 0.602 | |
|  | NPF+bert_AVE(FFNN) | 0.874 | 0.936 | 0.931 | 0.652 | 0.966 | 0.601 | |
|  | NPF+bert_AVE(BiLSTM) | 0.874 | 0.936 | 0.931 | 0.652 | 0.966 | 0.602 | |

**Table S6**. Performance comparison between the fine-tuned model of Promoter-BERT and the fine-tuned model of DNABERT.

|  | Model | Sen | Spe | ACC | MCC | AUROC | AUPRC |
| --- | --- | --- | --- | --- | --- | --- | --- |
| 5-fold cross validation | Promoter-BERT | 0.871 | 0.937 | 0.932 | 0.653 | 0.966 | 0.602 |
|  | DNABERT | 0.836 | 0.941 | 0.932 | 0.641 | 0.964 | 0.589 |
| Independent test | Promoter-BERT | 0.872 | 0.938 | 0.932 | 0.656 | 0.966 | 0.604 |
|  | DNABERT | 0.839 | 0.942 | 0.934 | 0.646 | 0.965 | 0.598 |
